# Pre-adolescence DNA methylation is associated with BMI status change from pre- to post-adolescence

**DOI:** 10.1186/s13148-021-01042-4

**Published:** 2021-03-25

**Authors:** Jiajing Wang, Hongmei Zhang, Faisal I. Rezwan, Caroline Relton, S. Hasan Arshad, John W. Holloway

**Affiliations:** 1 Division of Epidemiology, Biostatistics, and Environmental Health, School of Public Health, University of Memphis, Memphis, TN USA; 2 School of Water, Energy and Environment, Cranfield University, Cranfield, MK43 0AL Bedfordshire UK; 3 The David Hide Asthma and Allergy Research Centre, St Mary’s, Hospital, Parkhurst Road, Newport, PO30 5TG Isle of Wight UK; 4 Clinical and Experimental Sciences, Faculty of Medicine, University of Southampton, Southampton, SO16 6YD UK; 5 MRC Integrative Epidemiology Unit, University of Bristol, Bristol, UK; 6 Bristol Medical School, Population Health Sciences, University of Bristol, Bristol, UK; 7 Faculty of Medicine, Human Development and Health, University of Southampton, Southampton, SO16 6YD UK

**Keywords:** Adolescence, DNA methylation, Longitudinal, Body mass index (BMI), BMI status transition, Obesity

## Abstract

**Background:**

Previous studies have shown that DNA methylation (DNAm) is associated with body mass index (BMI). However, it is unknown whether DNAm at pre-adolescence is associated with BMI status transition from pre- to post-adolescence. In the Isle of Wight (IoW) birth cohort, genome-wide DNA methylation in whole blood was measured using Illumina Infinium Human450 and EPIC BeadChip arrays in n = 325 subjects, and pre- to post-adolescence BMI transition was classified into four groups: (1) normal to normal, (2) normal to overweight or obese, (3) overweight or obese to normal, and (4) persistent overweight or obese. We used recursive random forest to screen genome-wide Cytosine-phosphate-Guanine (CpG) sites with DNAm potentially associated with BMI transition for each gender, and the association of BMI status transition with DNAm at an earlier age was assessed via logistic regressions. To evaluate gender specificity, interactions between DNAm and gender were included in the model. Findings in the IoW cohort were further tested in an independent cohort, the Avon Longitudinal Study of Parents and Children (ALSPAC).

**Results:**

In total, 174 candidate CpGs were selected including CpGs from screening and CpGs previously associated correctionally with BMI in children and adults. Of these 174 CpGs, pre-adolescent DNAm of 38 CpGs in the IoW cohort was associated with BMI status transition, including 30 CpGs showing gender-specific associations. Thirteen CpGs showed consistent associations between the IoW cohort and the ALSPAC cohort (11 of which were gender-specific).

**Conclusion:**

Pre-adolescence DNAm is associated with the change in BMI status from pre- to post-adolescence and such associations are likely to be gender-specific.

**Supplementary Information:**

The online version contains supplementary material available at 10.1186/s13148-021-01042-4.

## Introduction

Adolescence, marked by onset of physiologically normal puberty between the ages of 10 and 19, is a transition phase of growth and development between childhood and adulthood [1]. During growth and maturation, body composition changes may be accompanied by dramatic changes in body mass index (BMI) and development of obesity [2–4]. In US children, the average BMI is 16.0 kg/${\mathrm{m}}^{2}$ at age 6 to 7 years, but by 16–17 years of age, average BMI is close to 22 kg/${\mathrm{m}}^{2}$ [4, 5]. A recent study showed a significant increase in BMI among males from age 12 to 19 years, but not among other age groups or among females [6]. In addition, national longitudinal data collected from US adolescents enrolled in wave II (1996) and wave III (2001) of the National Longitudinal Study of Adolescent Health showed that there was a high proportion of adolescents becoming and remaining obese into adulthood during a 5-year transition period between adolescence and young adulthood [7].

Both environmental [8–10] and genetic factors have been found to affect BMI and BMI change [11, 12]. The role of genetic and environmental factors interaction suggests that other gene regulatory mechanisms, such as epigenetic mechanisms, DNA methylation (DNAm) in particular, reflecting memory of past exposure or changes at different stages of life [13], may act as an interface between environment exposure and genetics. Various studies have contributed to the understanding of underlying epigenetic mechanisms or explore epigenetic markers of obesity or BMI. DNAm at certain CpG sites has been found to be associated with BMI during adolescence [14]. In an epigenome-scale study, DNAm at cg22891070 was shown to be inversely associated with expression of *HIF3A* in adipose tissue [15]. Recent meta-analyses also identified 187 CpG sites where DNAm was associated with BMI in adults [16] and 10 CpGs in children (age 2–10) and 1 CpG in adolescents (age 12–18) [17]. All these studies focused on the association between DNAm and BMI cross-sectionally at a single time point. DNAm reflects past environmental exposure, and thus potentially is on the casual pathway between early life environmental exposure and development of obesity in adolescence. Detecting early age epigenetic markers for BMI status change may benefit obesity early prevention and intervention. In this study, we hypothesized that at specific CpG sites, DNAm at an earlier age was associated with BMI status transition later in life.

## Results

In total, 325 children with BMI and DNAm available at both ages 10 and 18 years were included in the analyses. Among these 325 children, n = 186 (57.2%) were male (Table 1). Average ZBMIs and distribution of BMI status transitions for each gender were not statistically different from those in the complete cohort (*p* value > 0.05).

**Table 1:** Comparison between subsamples and complete samples in the IoW

	Subset sample (*n* = 325) *	Complete cohort (*n* = 806)	*p* value
**ZBMI (Mean ± SD)**			
*Age10*	0.45 (1.04)	0.29 (0.92)	0.45
Male	0.43 (1.01)	0.18 (0.92)	0.43
Female	0.49 (1.09)	0.39 (0.91)	0.49
*Age18*	0.53 (1.17)	0.20 (1.01)	0.53
Male	0.41 (1.16)	−0.03 (1.05)	0.41
Female	0.69 (1.18)	0.41 (0.93)	0.69
**N (%)**			
*Gender*			
Male	186 (57.2%)	382 (47.4%)	0.43
Female	139 (42.8%)	424 (52.6%)	
*BMI transition*			
Normal → OwO**	31 (10.8%)	68 (9.8%)	0.9
OwO → Normal	24 (8.3%)	66 (9.5%)	
OwO → OwO	30 (20.4%)	81 (11.7%)	
Normal → Normal	203 (70.5%)	479 (6.9%)	

^*^Subset samples are selected based on availability of DNA methylation data. ** OwO: Overweight/Obese

### Assessment of BMI status transition at previously identified BMI-associated CpGs

Of the 187 CpGs associated with BMI in adults in the study of Wahl et al. [16], 178 CpGs were available in the IoW cohort. Via linear mixed models, at 158 CpGs (88.8%), the associations of DNAm at age 10 years with BMI at 10 and 18 years in the IoW cohort had the same direction as those in Wahl et al. [16], and at 64 of these CpGs [see Additional file 1], the associations were statistically significant at the 0.05 level. These 64 CpGs from the Wahl et al.’s study [16], along with 10 of the 11 identified CpGs in Vehmeijer et al.’s study [17] (DNAm at 10 of the 11 CpGs were available in IoW), were further examined separately for their associations with BMI status transition across adolescence. There were no common CpGs between the 64 CpGs and the 10 CpGs.

Using a FDR = 0.05, one of the 64 CpG sites associated with cross sectional BMI in the IoW cohort was associated with BMI status transition across adolescence using logistic regressions; a higher methylation of cg07728579 in gene *FSD2* was associated with a higher odds of being in the transition from overweight / obese to normal group irrespective of gender (log odds ratio [log-OR] = 5.00, *p* value 3.38 × 10^–4^, Table 2). None of these 64 CpGs showed interaction effects with gender. Of the 10 CpGs that were identified in Vehmeijer et al.’s study [17], one CpG showed a statistically significant DNAm × sex interaction effect on BMI status transition across adolescence in the IoW cohort; at cg15125798, boys with higher DNAm were more likely to be in the transition group from normal weight to overweight or obese, but for girls, the association was in the opposite direction (*p* value for interaction effect: 2.3 × 10^–3^, Table 3).

**Table 2:** Effects of DNAm on BMI status transition (irrespective to gender) during adolescence

Name	10–18 years (IoW)			7–15 years (ALSPAC)			
	Est.*	*p*_raw_*	*P*_FDR_*	Est	*p*_raw_	Gene name	Gene’s location
Transition from Normal to OwO*							
cg14062083	6.49	1.29 × 10^–4^	0.01	–	–	*KRTAP13-4*	1stExon
**cg17866181**	−**2.94**	**2.76 × 10**^**–4**^	**0.01**	−**0.05**	**0.76**	***BC036251***	***Body***
Transition from OwO to Normal							
cg02906784	4.95	2.33 × 10^–4^	0.02	−0.56	0.31	*CACNA2D3*	*Body*
cg07728579	5.00	3.38 × 10^–4^	0.02	−0.33	0.50	*FSD2*	*TSS500*
cg18935453	5.08	5.79 × 10^–4^	0.02	–	–	*LRP5*	*Body*
cg23664926	2.76	7.16 × 10^–4^	0.02	−0.11	0.72	*CD82*^*#*^	–
cg10320997	3.83	9.97 × 10^–4^	0.02	−0.17	0.67	*SYNPO2*	*TSS1500*
cg02382044	2.30	0.002	0.04	−0.44	0.20	*TMOD3*	*TSS1500*
**cg16460342**	−**3.63**	**0.002**	**0.04**	−**0.65**	**0.07**	***P2RX4***	**Body**

In total, 9 CpG sites were identified in the IoW cohort, which were further tested in the ALSPAC cohort. Results in bold font denote that the estimated regression coefficients are in the same direction between the two cohorts

^*^OwO means Overweight/Obese. Est.: Estimate of regression coefficients. p_raw_: raw *p* value. p_FDR_: FDR-adjusted *p* value. # Gene *CD82* was identified using SNIPPER

**Table 3:** Gender specificity in the effects of DNAm on BMI status transition during adolescence

Name	10–18 years (IoW)					7–15 years (ALSPAC)					
	Main effects (DNAm)		Interaction effects (DNAm** × **gender)			Main effects (DNAm)		Interaction effects (DNAm** × **gender)		Gene name and Gene’s location	
	Est.*	*p*_raw_*	Est	*p*_raw_	*p*_FDR_*	Est	*p*_raw_	Est	*p*_raw_	Name	Location
	Persistence of OwO*										
cg15648188	2.86	0.02	−6.72	2.89 × 10^–4^	0.03	0.63	0.24	0.08	0.91	*BC023516*	*Body*
cg04131949	−2.22	0.03	5.83	1.60 × 10^–3^	0.04	0.28	0.46	−0.89	0.12	*RAB11FIP3*	*Body*
cg25806347	2.16	0.24	−11.05	2.43 × 10^–3^	0.04	−0.15	0.77	−0.16	0.82	*SIX2*	*Body*
cg24982697	−5.07	4.89 × 10^–4^	5.46	2.56 × 10^–3^	0.04	0.29	0.32	−0.26	0.54	*ATP11A*	*Body*
cg02746620	−8.06	6.44 × 10^–4^	8.89	8.21 × 10^–4^	0.04	0.20	0.71	0.01	0.99	*SLIT3*	*Body*
cg13585675	−6.21	0.002	7.24	0.002	0.04	0.17	0.66	−0.35	0.56	*NPTX2*	*Body*
cg16460342	1.09	0.29	−5.62	2.62 × 10^–3^	0.04	−0.22	0.60	0.03	0.96	*P2RX4*	*Body*
**cg00283283**	−**11.47**	**0.001**	**12.71**	**3.61 × 10**^**–3**^	**0.04**	−**0.35**	**0.53**	**0.27**	**0.75**	***PCDHGB5;PCDHGC3;PCDHGA6***	***3′UTR;3′UTR;3′UTR;3′UTR;3′U***
**cg00928795**	−**6.33**	**0.01**	**9.11**	**3.80 × 10**^**–3**^	**0.04**	−**0.33**	**0.58**	**0.33**	**0.71**	***AK057218***	***Body***
cg02380833	3.49	0.02	−5.13	5.00 × 10^–3^	0.05	−0.65	0.16	0.75	0.25	*PTPRO*	*TSS1500*
	Transition from Normal to OwO										
cg01959050	−6.12	6.76 × 10^–4^	10.21	6.69 × 10^–5^	0.007	0.15	0.85	−0.23	0.81	*DKFZP451C023*	*Body*
**cg15511327**	**3.06**	**0.04**	−**9.24**	**1.83 × 10**^**–4**^	**0.01**	**0.30**	**0.58**	−**0.26**	**0.70**	***LYNX1***	***1stExon;5′UTR;TSS1500;1stEx***
**cg07719077**	−**2.35**	**0.17**	**8.01**	**5.56 × 10**^**–3**^	**0.04**	−**0.26**	**0.66**	**0.04**	**0.96**	***KIAA1211***	***Body***
cg14478163	7.24	0.002	−8.08	2.35 × 10^–3^	0.04	−0.59	0.33	0.25	0.74	*ANKRD34B*	*TSS200*
cg21862373	5.87	0.009	−7.48	5.07 × 10^–3^	0.04	−1.41	0.09	1.51	0.12	*SNHG9;RPS2;SNORA78*	*TSS1500;5′UTR;TSS1500*
cg14610958	−2.75	0.007	3.86	4.38 × 10^–3^	0.04	0.03	0.97	−0.60	0.43	*ANGEL2*	*TSS1500*
**cg00331210**	−**8.60**	**0.009**	**12.21**	**1.96 × 10**^**–3**^	**0.04**	−**0.24**	**0.74**	**0.78**	**0.37**	***NARFL***	***3′UTR***
cg22059735	−1.13	0.44	6.15	4.75 × 10^–4^	0.04	–	–	–	–	*AK057493;BC043255;BC035866*	*Body*
**cg07378760**	−**0.72**	**0.08**	**2.36**	**2.63 × 10**^**–3**^	**0.04**	−**0.77**	**0.65**	**0.74**	**0.69**	***FLNB***	***TSS200***
cg24158437	−7.02	0.002	7.23	3.82 × 10^–3^	0.04	1.07	0.10	−0.99	0.20	*KLHL32*	*Body*
cg13629132	−3.71	0.009	4.86	5.26 × 10^–3^	0.04	0.62	0.20	−0.88	0.11	*AX747623*	*Body*
cg08402439	−3.78	0.03	7.81	1.60 × 10^–3^	0.04	0.25	0.57	−0.61	0.27	*AGA;NEIL3*	*Body*
**cg04120329**	**3.70**	**6.73 × 10**^**–4**^	−**3.85**	**3.89 × 10**^**–3**^	**0.04**	**0.67**	**0.20**	−**0.88**	**0.16**	***PRAP1***	***TSS1500***
cg11878331	0.86	0.07	−2.43	4.94 × 10^–3^	0.04	−0.32	0.71	0.34	0.76	*BC048982*	*Body*
**cg23244169**	**0.45**	**0.68**	−**4.71**	**6.23 × 10**^**–3**^	**0.04**	**0.43**	**0.45**	−**1.18**	**0.09**	***C17orf45***	***Body***
cg00842549	5.83	6.32 × 10^–4^	−5.84	7.76 × 10^–3^	0.05	0.18	0.74	0.32	0.63	*HOXB3*	*TSS200*
**cg15125798**	−**2.41**	**0.03**	**4.52**	**2.30 × 10**^**–3**^	**0.02**	−**0.52**	**0.32**	**0.25**	**0.68**	***–***	***–***
	Transition from OwO to Normal										
cg11249998	6.64	0.003	−9.95	3.83 × 10^–4^	0.01	−0.04	0.96	0.10	0.93	*NME2;NME2;NME1-NME2;NME*	*5′UTR;TSS1500;Body;1stExon;*
**cg15708019**	**2.66**	**0.003**	−**3.81**	**2.42 × 10**^**–4**^	**0.01**	**0.09**	**0.95**	−**0.69**	**0.61**	***WDR19***	***TSS200***
**cg14260083**	**-2.65**	**0.009**	**5.11**	**4.13 × 10**^**–4**^	**0.01**	**-0.19**	**0.58**	**1.22**	**0.04**	***RADIL***	***Body***

In total, 29 CpGs were identified in the IoW cohort and tested in the ALSPAC cohort. Female is the reference group. Results in bold font denote that the estimated regression coefficients (both main effects and interaction effects) are in the same directions between the two cohorts

^*^OwO means Overweight/Obese. Est.: Estimate of regression coefficients. *p*_raw_: raw *p* value. *p*_FDR_: FDR-adjusted *p* value.

### Assessment of BMI status transition at candidate CpGs identified via recursive Random Forest

In total, 100 of the 439,586 CpGs were selected based on results from the recursive random forest machine-learning technique based on non-parametric associations of DNAm at age 10 years with BMI status transition. After controlling FDR = 0.05, at 36 of these 100 CpGs, DNAm at ages 10 years was significantly associated with BMI status transition from 10 to 18 years of age. These 36 CpGs included 8 CpGs where the association was gender independent, and 29 CpGs (with one CpG site, cg16460342, also among the 8 main-effect-only CpGs) with gender-specific associations.

Among the 8 CpG sites with DNAm at age 10 years associated with the transition of BMI status between 10 and 18 (Table 2), regardless of gender, at 5 CpG sites (Table 2), subjects with higher DNAm were more likely to be in the transition to normal group (from obese or overweight to normal. The estimated log-OR were all > 2 with *p* values ≤ 2 × 10^–3^. Table 2). At the other 3 CpGs, pre-adolescence DNAm was associated with transition from overweight/obese to normal but in an opposite direction compared to the aforementioned 5 CpGs (cg16460342, log-OR = -3.63, *p* value = 0.002) or with transition from normal weight to obese or overweight (cg14062083 and cg17866181, *p* value < 3 × 10^–4^. Table 2).

Statistically significant interaction effects were identified between DNAm at age 10 and BMI status transition across adolescence at 29 CpG sites (Table 3). At 10 of these 29 CpGs, DNAm at age 10 years was associated with the persistence of overweight/obesity across adolescence, and the associations were opposite between boys and girls. Specifically, at 4 CpGs, for girls, subjects with higher DNAm were more likely to have persistent obese or overweight, but for boys, the associations were in the opposite direction (*p* values for interaction effects < 5 × 10^–3^). At the other 6 CpGs (Table 3), a higher DNAm was associated with higher odds of being persistent obese or overweight for boys but the opposite direction of associations was observed for girls (*p* values for interaction effects < 0.002).

DNAm at 16 of the 29 CpGs showed statistically significant gender-specific associations with BMI status transition from normal weight to overweight or obese (Table 3). At 7 of these 16 CpG sites, among girls, higher DNAm at age 10 years was associated with higher odds of becoming overweight or obese in adolescence, but for boys, the association was opposite (*p* values for interaction effects < 8 × 10^–3^. Table 3). At the remaining 9 of the 16 CpG sites, girls with higher DNAm at age 10 years were less likely to be in the transition to overweight or obesity group, but for boys the transition was more likely (*p* values for interaction effects < 6 × 10^–3^). For the remaining three of the 29 CpGs (cg11249998, cg15708019, cg14260083), DNAm at age 10 years showed gender-specific associations with BMI status transition from being overweight or obese to normal weight (all *p* values < 4 × 10^–4^. Table 3).

### Replication in ALSPAC

The 38 CpG sites associated with BMI status transition across adolescence (including one CpG from Wahl et al. [16], one from Vehmeijer et al. [17], and 36 from the candidate CpGs using epigenome-wide screening in the IoW cohort) were further assessed in the ALSPAC cohort. Consistent regression coefficients that were statistically significant or marginally significant at the 0.05 level were shown in 2 of the 38 CpGs in ALPSAC, including one gender-unspecific CpG site cg16460342 (*P2RX4*, *p* value = 0.07), and one gender-specific CpG, cg14260083 (*RADIL*, *p* value = 0.04). Compared to other CpG sites, larger interaction effects were observed in both cohorts at cg14260083; girls with a higher level of DNAm at this CpG site were less likely in the transition to normal weight group, but boys were more likely to be in this group if they had a higher level of DNAm (Table 3). In addition, although not statistically significant, at another 11 of the 38 CpGs, consistent directions of associations were observed either in main effects (Table 2) or in interaction effects (Table 3). Thus, in total at 13 of the 38 CpG sites, consistent associations were observed between the two cohorts, IoW and ALSPAC.

### Pathway analysis

In total, 13 CpGs showed consistent effects between the IoW and ALSPAC cohorts. The 13 CpGs were mapped to 14 genes in total (Tables 2 and 3). Six pathways and eight processes were detected using ToppFun after controlling FDR of 0.05 with pathways involving at most 2000 genes. Among the 14 genes, eight genes were present in these pathways or processes, including *FLNB*, *LYNX1*, *P2RX4*, *RADIL*, *WDR19*, and three genes in the *PCDHG* family (Table 4).

**Table 4:** Biological pathways/processes on genes with CpGs showing consistent interaction effects between IoW and ALSPAC

Gene		Source	*p* values*
	Pathways	Pathways	
*P2RX4*	REACTOME	Elevation of cytosolic Ca2 + levels	9.521 × 10^–3^
		Platelet calcium homeostasis	1.730 × 10^–2^
*WDR19*		Intraflagellar transport	2.283 × 10^–2^
*FLNB*		Antiviral mechanism by IFN-stimulated genes	4.251 × 10^–2^
		*ISG15* antiviral mechanism	4.251 × 10^–2^
*FLNB*	KEGG	Salmonella infection	4.737 × 10^–2^
		GO biological processes	
*WDR19*		Myotome development	1.588 × 10^–3^
		Ciliary receptor clustering involved in smoothened signaling pathway	1.588 × 10^–3^
*PCDHGB5, PCDHGC3, PCDHGA6, RADIL*		Cell adhesion	1.718 × 10^–3^
*P2RX4*		Positive regulation of microglial cell migration	1.984 × 10^–3^
		Sensory perception of touch	2.381 × 10^–3^
		Regulation of microglial cell migration	2.777 × 10^–3^
*P2RX4, LYNX1*		Chemical synaptic transmission, postsynaptic	2.589 × 10^–3^
*FLNB*		Cytoskeletal anchoring at plasma membrane	4.756 × 10^–3^

^*^The *p* values are multiple testing adjusted by controlling a false discovery rate of 0.05

### Biological relevance assessment

For the 13 CpG sites showing consistent associations between IoW and ALSPAC cohorts, expression data at 7 mapped genes (corresponding to 6 CpGs) were available. At 4 of the 6 CpGs, the association of DNAm with expression of their mapped genes was statistically significant or marginally significant at a significance level of 0.05. Furthermore, at 3 of those 4 CpGs, DNAm was inversely correlated with expression of genes; for instance, at cg00283283 (on gene *PCDHGA6*), the Spearman’s correlation coefficient is ρ = −0.269 (*p* value = 0.001), indicating higher DNAm was associated with lower gene expression (Table 5).

**Table 5:** Correlation between DNAm and gene expression for CpGs showing associations consistent between the IoW and ALSPAC cohorts

CpG sites	Gene	Correlation	*p* values*
**cg16460342**	***P2RX4***	**0.208**	**0.014**
**cg00283283**	*PCDHGC3*	−0.034	0.690
	***PCDHGA6***	−**0.269**	**0.001**
cg15511327	*LYNX1*	−0.156	0.066
cg07719077	*KIAA1211*	−0.166	0.051
cg00331210	*NARFL*	−0.013	0.883
cg04120329	*PRAP1*	0.084	0.324

Only CpGs with available expression data of their mapped genes are included in the table

^*^Results in bold font had *p* value < 0.05, and those underlined had *p* value close to 0.05

## Discussion

The aim of this study was to examine the association of pre-adolescent DNAm with BMI status transition during adolescence. Among the 38 CpGs identified in the IoW cohort, consistent associations were observed in the ALSPAC cohort at 13 CpGs and in 11 of these 13 CpGs, the associations were gender specific. In two of the 13 CpGs (cg16460342 in the body of *P2RX4* and cg14260083 in the body of *RADIL*), the associations (main effects or interaction effects) were statistically significant or marginally significant in both cohorts. Most of the CpGs (4 of 6 CpGs) included in the assessment for their association with gene expression indicated a potential of biological relevance.

Among the pathways identified, although not all the identified pathways or biological processes are involved in the development of obesity, several pathways or processes drew our attention. In the pathway of elevation of cytosolic Ca2 + levels and platelet calcium homeostasis, a recent study indicated that inflammation, either induced by cytokine exposure in vitro or by obesity in vivo, led to increased expression and activity of *IP3Rs* in adipocytes in a JNK-dependent manner and then increased cytosolic Ca2 + and impaired insulin action [18]. Another study also showed that weight loss restored *SERCA3* activity and subsequent calcium signaling, αIIbβ3 activation, platelet aggregation, and ADP secretion [19]. In the pathway of antiviral mechanism by IFN-stimulated genes *ISG15* antiviral mechanism, interferons activated JAK–STAT signaling, which led to the transcriptional induction of hundreds of IFN-stimulated genes (ISGs). The ISG proteins generated by IFN pathways included direct effectors which inhibit viral infection through diverse mechanisms as well as factors that promote adaptive immune responses [20]. Antiviral interferons (IFNs), as key immune regulators against viral infections and in autoimmunity, emerged to be a pivotal player in the regulation of adipogenesis [21]. In addition, for the GO biological process of cell adhesion, a recent study found that LSECs were involved in obesity-associated accumulation of myeloid cells via VLA-4-dependent cell–cell adhesion [22]. A better understanding of these pathways and processes from the epigenetic perspective may benefit obesity prediction and prevention at an earlier age.

The eight genes present in the identified pathways or biological processes include *P2RX4* and *RADIL*, both these genes have been shown to be associated with obesity or BMI. Gene *P2RX4* is a protein coding gene and the receptor *P2X4* can be found in uterine endometrium and fat cells, and in the smooth muscle of arteries [23]. Obesity is characterized by immune cell infiltration and inflammation. A recent study by Ruíz-Rodríguez et al. shows that an increase in *P2XR* receptor expression could be associated with a progression in the metabolic state and the progression in the metabolic state is associated with an increase in BMI [24]. Rather than looking at one time point, our study focused on BMI status transition. Together with findings in Ruiz-Rodriguez et al. [24], our results from epigenetic and gene expression analyses supported that subjects with lower DNAm at cg16460342 on *P2RX4* at pre-adolescence are more likely to experience a transition from normal weight to overweight or obese, rather than stay at normal weight through adolescence.

DNAm of cg14260083 on gene *RADIL* showed a gender-specific association with BMI status transition accompanied by large interaction effects observed in both IoW and ALSPAC cohorts. For females, our study showed that subjects with increased DNAm at this CpG site were less likely to be in the transition from overweight or obese to normal group, but for males, they were more likely. Evidence is limited on the direct connection between *RADIL* and BMI or obesity, but an earlier study demonstrated that gene *RADIL* was significantly associated with birthweight [25] and birth weight has been shown to be linked to obesity [26, 27]. Gender-specific effects of an epigenetic site in gene *RADIL* on BMI status transition as shown in our study have not been discussed in the literature.

The two genes (*P2RX4* and *RADIL*), identified in the IoW and further confirmed in the ALSPAC, had some limited evidence of connections with BMI or obesity as discussed above. Although further investigation may be needed, the limited findings of these genes on their connection with BMI implies that the underlying epigenetic mechanisms associated with BMI and those linked to BMI status transition were likely to be different. Consequently, our findings indicate that the identified two CpGs on these two genes are likely to be informative biomarkers for BMI status transition during adolescence. For their potential to behave as causality epigenetic markers, further in-depth assessment is warranted.

It is worth noting that among the 74 candidate CpGs selected from Wahl et al. [16] and Vehmeijer et al. [17], only 2 CpGs (2.7%) were shown to be associated with BMI status transition in the IoW cohort. This is as expected. Both the studies of Wahl et al. and Vehmeijer et al. (the assessment in children) were cross sectional and, as such, the methylation of CpGs identified is more likely to be a consequence of BMI change, as shown in both studies. The present study, in contrast, aimed to identify potential early epigenetic markers for future BMI status change. Thus, the findings from our study did not conflict with those in Wahl et al. [16] and Vehmeijer et al.[17]; we examined the connection of DNAm with BMI-related outcomes (i.e., BMI status change) from a different angle. We would like to point out, however, that there is a possibility that our identified CpGs were due to methylation quantitative trait loci (methQTLs), which deserves further assessment.

Gender-specificity in the associations of pre-adolescence DNAm with BMI status transition was observed in most identified CpGs. At 13 CpGs showing consistent associations between the IoW cohort and the ALSPAC cohort, gender-specificity was shown at 11 of the 13 CpGs (> 80%), indicating a possibility of different epigenetic driving factors of BMI status transition between males and females. It is worth noting that these 13 CpGs represent a relatively small portion (~ 30%) of CpGs identified in the IoW cohort. It was noted that ZBMIs in males and females disagreed on average between the two cohorts for each gender (Additional file 2). In addition, DNA methylation in the IoW cohort was measured at ages 10 and 18 years, while in the ALSPAC cohort, the ages are 7 and 15 or 17 years. At the age of 15 years, children might be still undergoing adolescence transition. All these might have influenced the degree of consistency in findings between the two cohorts. Further large-scale epigenetic studies are warranted to thoroughly evaluate the findings and establish their validity.

## Conclusion

In conclusion, we demonstrate that DNAm at pre-adolescence is associated with BMI status transition from pre- to post-adolescence and the associations are likely to be gender-specific. The identified CpGs have the potential to serve as candidate epigenetic factors in future studies with focus on epigenetic mechanisms of obesity in adolescence.

## Methods

### Study population, the Isle of Wight cohort

An unselected whole population birth cohort study was established in 1989/1990 on the Isle of Wight (IoW), UK [28]. After exclusion of adoptions, perinatal deaths and refusal for follow-up, 1,456 were enrolled and the study collected children’s information at ages 1 or 2, 4, 10, 18, and 26 years. The study was approved by the Isle of Wight Local Research Ethics Committee, and written informed consent was obtained from parents, participants, or both. Data from participants with measurements of height and weight at both ages, puberty onset age, and personal smoking status were collected. BMI z-scores (ZBMI) were calculated based on the British standards [29] for children at ages 10 and 18 years.

### DNA methylation (DNAm)

DNA was extracted from whole blood samples collected at ages 10 and 18 years using a standard salting out procedure [30]. DNAm was measured using the Illumina Infinium HumanMethylation450 and MethylationEPIC Beadchips (henceforth will be denoted as 450 k and EPIC, respectively) (Illumina, Inc., San Diego, CA, USA), which interrogate > 484,000 and > 850,000 CpG sites, respectively. Data pre-processing was undertaken using the CPACOR pipeline [31]. Briefly, intensity values were background corrected and assessed for quality. Probes not reaching a detection *p* value of ${10}^{-16}$ in at least 95% of samples were excluded. The data were quantile normalized using the R package, *minfi* [32]. Autosomal probes were then extracted and methylation level was converted to β values. Principal components (PCs) inferred based on control probes were used to represent latent chip-to-chip and technical variations. Since DNAm data were from two different platforms, we determined the PCs based on DNAm at shared control probes. In total, 195 control probes were shared between the two arrays and used to calculate the control probe PCs with the top 15 to represent latent batch factors [33]. CpG sites common between the Illumina 450 k and EPIC platforms were included in this study. To reduce the potential influence of probe SNPs, CpG sites were further excluded if a SNP was within 10 base pairs of the targeted CpG site and if the minor allele frequency in the Caucasian population was > 7%. After pre-processing, in total, 439,586 CpGs were included for analyses.

Blood is a mixture of functionally and developmentally distinct cell populations [34] and adjusting for cell-type compositions removes potential confounding effects of cell heterogeneity in DNAm in blood samples [35]. Cellular composition of the blood sample was calculated using function e*stimateCellCounts* in the R package *minfi* [36, 37]. The proportions of six cell types, CD8T, CD4 + T cells, natural killer cells, B cells, monocytes, and granulocytes cells were estimated. At each of the 439,586 CpG sites, logit transformed DNAm at base 2 was regressed on the 15 PCs and the proportions of the cell types (except for CD8T to avoid collinearity), and the residuals were batch- and cell type-adjusted DNAm, for inclusion in subsequent analyses.

### Genome-wide RNA-seq gene expression data generation

Gene expression levels from peripheral blood samples collected at 26 years from the IOWBC were determined using paired-end (2 × 75 bp) RNA sequencing with the Illumina Tru-Seq Stranded mRNA Library Preparation Kit with IDT for Illumina Unique Dual Index (UDI) barcode primers following the manufacturer’s recommendations. All samples were sequenced twice using the identical protocol, and for each sample, the output from both runs was combined. FASTQC were run to assess the quality of the FASTQ files (https://www.bioinformatics.babraham.ac.uk/projects/fastqc/). Reads were mapped against Human Genome (GRch37 version 75) using HISAT2 (v2.1.0) aligner [34]. The alignment files, produced in the Sequence Alignment Map (SAM) format, were converted into the Binary Alignment Map (BAM) format using SAMtools (v1.3.1) [35]. HTseq (v0.11.1) was used to count the number of reads mapped to each gene in the same reference genome used for alignment [36]. Normalized read count FPKM (Fragments Per Kilobase of transcript per Million mapped reads) were calculated using the countToFPKM package (https://github.com/AAlhendi1707/countToFPKM), and the log-transformed values were used for data analysis. In this study, RNAseq gene expression data of n = 304 subjects with DNAm at age 10 years also available were included in the analyses for biological relevance at identified CpG sites.

### Statistical analysis

The study samples were compared with the whole cohort using one sample t-tests for continuous variables, one sample proportion tests for percentages, and Chi-square tests for categorical variables. A *p* value < 0.05 was considered as being statistically significant. To identify candidate CpG sites potentially associated with BMI status transition, two approaches were used: assessment of previously identified BMI-associated CpG sites, and epigenome-wide screening in the IoW cohort.

### Previously identified BMI-associated CpG sites

In a previous epigenome-wide association study (EWAS) of 5387 individuals in multiple cohorts, Wahl et al. [16] identified DNAm of 187 CpGs associated with BMI in adults (mean age > 50 years). To assess the longitudinal relationship between each of these CpGs and BMI in adolescents in the IoW cohort with random subject effects addressed, linear mixed models were implemented in which DNAm at ages 10 and 18 years was the dependent variable and BMI at ages 10 and 18 years, gender and exposure to smoke evaluated at ages 10 and 18 years were independent variables. Those CpG sites associated with BMI in the IoW cohort (*p* value < 0.05) were used in subsequent analyses to examine their association with BMI status transition across adolescence. In another EWAS assessment with 4133 children from 23 studies, Vehmeijer et at. [17] identified 11 CpGs that associated with BMI in the age from 2 to 18 years based on cross-sectional analyses. We also included these 11 CpGs in our study and assessed their association with BMI status transition.

### Epigenome-wide CpG screening in the IoW cohort

A recursive random forest [38, 39] algorithm in the R package *RandomForest* was applied to screen CpG sites where DNAm at age 10 years was associated with ZBMI status transition from ages 10 to 18 years at each gender. CpGs that passed screening were included in subsequent analyses. For each gender, the top 50 CpG sites that reduced the mean Gini indices to the maximum extent were included in subsequent analyses.

### DNAm at pre-adolescence with BMI status transition during adolescence

BMI status transition was defined based on ZBMIs. A child with ZBMI ≥ 2 was considered obese, between 1.333 and 2 overweight, between −2.667 and −2 thinness, and ≤ -2.667 severe thinness. Otherwise, the child’s BMI was regarded as being in the normal range. Four BMI transition statuses between ages 10 and 18 years were examined in our study: (1) normal to normal, (2) transition from normal to overweight or obese, (3) transition to normal defined as changing from overweight or obesity to normal, and (4) persistence defined as subjects persistently overweight or obese from pre- to post-adolescence. Logistic regressions were applied to evaluate the odds of BMI status transition with normal to normal transition as the reference. Gender, age of puberty onset, personal smoking status, and maternal BMI were included as confounders. To evaluate whether the effect of DNAm was gender-specific, the model was further extended by including the interaction effect between gender and DNAm at each CpG site. A *p* value < 0.05 after controlling false discovery rate (FDR) of 0.05 was considered as being statistically significant.

### Further assessment in an independent cohort

The findings in the IoW cohort were tested in an independent cohort, the Avon Longitudinal Study of Parents and Children (ALSPAC). Details of the cohort were described elsewhere [40–42]. Women residing in the South West of England who were pregnant and expecting to deliver between April 1, 1991, and December 31, 1992, were eligible to be recruited. Of the 14,541 pregnant women eligible for recruitment, 13,761 were included in the study with 10,321 participants having their DNA sampled. DNAm was assessed using the Infinium HumanMethylation450 BeadChip. Please note that the study website contains details of all the data that is available through a fully searchable data dictionary and variable search tool (http://www.bristol.ac.uk/alspac/researchers/our-data/).

DNAm data from 1,018 offspring in the ALSPAC cohort were available at ages 7 and 15 or 17 years. The pre-processing of DNAm was performed by correcting for batch effects using the *minfi* package [32] and removing CpGs with detection *p* value ≥ 0.01. Samples were flagged that contained sex-mismatch based on X-chromosome methylation. Proportions of the same six cell types were estimated. The batch- and cell type-adjusted DNAm was estimated as for the IoW cohort.

ZBMI transition of a child from ages 7 to 15 or 17 years was inferred in the same way as that in the IoW cohort. Those CpGs showing regression coefficients consistent with those in the IoW cohort and a corresponding *p* value < 0.05 at marginally significant were treated as successfully replicated CpGs.

### Pathway analysis

The genes corresponding to the identified CpGs were annotated using the Illumina array manifest gene annotations and SNIPPER (https://csg.sph.umich.edu/ boehnke/snipper/) version 1.2, and the UCSC Genome Browser on Human Mar.2006 Assembly. ToppFun (https://toppgene.cchmc.org/enrichment.jsp) was applied to detect functional enrichment of pathways and biological processes using KEGG and REACTOME pathways and Gene Ontology (GO) biological processes. An adjusted *p* value < 0.05 after controlling FDR was considered as being statistically significant.

### Biological relevance of identified CpGs

For CpG sites showing consistent associations with BMI status change between the IoW and ALSPAC cohorts, we examined their biological relevance by assessing the association of age 10 DNAm at a CpG site with expression of its mapped genes at age 26 years. Spearman’s rank correlation was used to assess the associations. Statistical significance was inferred at *p* value < 0.05.

## Supplementary Information

**Additional file 1.** This additional file contains results in the IoW cohort from linear mixed models of the 64 previously identified BMI-associated CpGs.

**Additional file 2.** This additional file contains the comparison of ZBMIs between IoW and ALSPAC cohort.

## Data Availability

Data from the Isle of Wight birth cohort used and/or analyzed during the current study are available from the corresponding author on reasonable request. Data from the ALSPAC cohort that support the findings of this study are available from the UK Medical Research Council and Wellcome (Grant ref: 217065/Z/19/Z) and the University of Bristol provide core but restrictions apply to the availability of these data, which were used under license for the current study, and so are not publicly available.

## Abbreviations

DNAm: DNA methylation
BMI: Body mass index
IoW: Isle of Wight
CpG: Cytosine-phosphate-Guanine
ZBMI: BMI z-scores
PCs: Principal components
EWAS: Epigenome-wide association study
ALSPAC: Avon Longitudinal Study of Parents and Children
Log-OR: Log odds ratio
