# Supplementary material for: Pre-adolescence DNA methylation is associated with BMI status change from pre- to post-adolescence
Source: Clin Epigenetics. 2021 Mar 25;13:64. doi: 10.1186/s13148-021-01042-4 (PMC7995693; doi:10.1186/s13148-021-01042-4)
Supplement: Supplementary file 1 — Additional file 1. This additional file contains results in the IoW cohort from linear mixed models of the 64 previously identified BMI-associated CpGs. [file 13148_2021_1042_MOESM1_ESM.docx]

Table 1: Results were from linear mixed models with repeated measures.

| CpG | Gene | Coefficient | Raw P |
| --- | --- | --- | --- |
| cg06500161 | *ABCG1* | 0.009 | 2.05×10^-9^ |
| cg07728579 | *FSD2* | 0.008 | 4.43×10^-5^ |
| cg11024682 | *SREBF1* | 0.005 | 1.79×10^-4^ |
| cg01798813 | *ZZEF1* | 0.013 | 1.81×10^-4^ |
| cg11927233 | *NPM1* | 0.010 | 4.47×10^-4^ |
| cg00138407 | *KLHL18* | 0.007 | 4.77×10^-4^ |
| cg08548559 | *PIK3IP1* | -0.011 | 5.76×10^-4^ |
| cg10927968 | *MOB2;CTSD* | 0.011 | 6.35×10^-4^ |
| cg07136133 | *PRR5L* | -0.008 | 6.36×10^-4^ |
| cg08726900 | *ANKRD11* | -0.010 | 6.83×10^-4^ |
| cg01243823 | *NOD2* | -0.009 | 1.02×10^-3^ |
| cg27184903 | *APBA2* | 0.005 | 1.36×10^-3^ |
| cg27614723 | *SLCO3A1* | 0.004 | 1.86×10^-3^ |
| cg24174557 | *TMEM49* | -0.008 | 2.02×10^-3^ |
| cg03318904 | *MAP3K7IP1* | 0.007 | 2.26×10^-3^ |
| cg13123009 | *LY6G6E;LY6G6D;LY6G6* | 0.004 | 2.83×10^-3^ |
| cg01101459 | *LINC00184;IRF2BP2* | 0.006 | 3.37×10^-3^ |
| cg23232188 | *EAF2* | 0.005 | 3.63×10^-3^ |
| cg06192883 | *MYO5C* | 0.005 | 4.38×10^-3^ |
| cg22103219 | *SH2B2* | -0.005 | 5.52×10^-3^ |
| cg00973118 | *AXIN1* | 0.005 | 5.96×10^-3^ |
| cg03957124 | *FGD2* | -0.004 | 6.01×10^-3^ |
| cg25001190 | *NFIA* | -0.008 | 6.41×10^-3^ |
| cg06946797 | *RMI2* | -0.007 | 6.89×10^-3^ |
| cg02716826 | *SUGT1P1;AQP3* | -0.005 | 8.14×10^-3^ |
| cg11376147 | *SLC43A1* | -0.003 | 8.23×10^-3^ |
| cg24403644 | *TOX2* | 0.004 | 8.58×10^-3^ |
| cg14476101 | *PHGDH* | -0.007 | 1.04×10^-2^ |
| cg10179300 | *TRIO* | 0.007 | 1.05×10^-2^ |
| cg04577162 | *RFC2* | 0.005 | 1.09×10^-2^ |
| cg11832534 | *WDR8* | 0.009 | 1.18×10^-2^ |
| cg15357118 | *UGGT1* | 0.004 | 1.45×10^-2^ |
| cg08813944 | *CPSF4L* | 0.009 | 1.55×10^-2^ |
| cg27115863 | *CARD10* | -0.004 | 1.56×10^-2^ |
| cg08309687 | *ATP5PO* | -0.006 | 1.68×10^-2^ |
| cg08857797 | *VPS25* | 0.004 | 1.77×10^-2^ |
| cg17901584 | *DHCR24* | -0.005 | 1.77×10^-2^ |
| cg06012428 | *ARID1B;ARID1* | -0.004 | 1.80×10^-2^ |
| cg02286155 | *SLC34A1* | 0.003 | 1.91×10^-2^ |
| cg19750657 | *UFM1* | 0.005 | 2.05×10^-2^ |
| cg19373099 | *MAP2;PTH2R* | 0.008 | 2.11×10^-2^ |
| cg11969813 | *P4HB* | 0.006 | 2.11×10^-2^ |
| cg00634542 | *SLC11A1* | 0.005 | 2.23×10^-2^ |
| cg09554443 | *CD247* | -0.005 | 2.26×10^-2^ |
| cg22534374 | *RPS10P7;CSRP1* | -0.006 | 2.26×10^-2^ |
| cg10922280 | *DPEP2* | 0.004 | 2.59×10^-2^ |
| cg17178175 | *NFE2L2;NFE2L* | -0.005 | 2.61×10^-2^ |
| cg21486834 | *RHBDF2* | 0.004 | 2.76×10^-2^ |
| cg10505902 | *PDE4DIP;PDE* | -0.005 | 2.97×10^-2^ |
| cg25217710 | *BCAN* | 0.003 | 2.97×10^-2^ |
| cg26804423 | *ICA1* | 0.004 | 3.01×10^-2^ |
| cg10919522 | *C14orf43* | -0.005 | 3.02×10^-2^ |
| cg17501210 | *RPS6KA2* | -0.005 | 3.30×10^-2^ |
| cg13922488 | *PKN1* | 0.003 | 3.35×10^-2^ |
| cg11202345 | *LGALS3BP* | 0.006 | 3.39×10^-2^ |
| cg02711608 | *SLC1A5,SLC1A* | -0.004 | 3.50×10^-2^ |
| cg27547344 | *TIE1* | 0.003 | 3.63×10^-2^ |
| cg07037944 | *DAPK2* | -0.004 | 3.98×10^-2^ |
| cg03940776 | *SYNJ2* | -0.002 | 4.06×10^-2^ |
| cg08648047 | *C1orf127* | 0.004 | 4.28×10^-2^ |
| cg07769588 | *ATG4D* | 0.004 | 4.56×10^-2^ |
| cg22695339 | *CHD3* | -0.004 | 4.67×10^-2^ |
| cg09152259 | *PROC* | -0.004 | 4.69×10^-2^ |
| cg26687842 | *LOC646982* | 0.004 | 4.73×10^-2^ |
| Note: These CpGs show the same directions of association with BMI as in Wahl et al. (2017) and were statistically significant at the 0.05 significance level, using data in the IoW cohort. | | | |
