# Supplementary material for: Pre-adolescence DNA methylation is associated with BMI status change from pre- to post-adolescence
Source: Clin Epigenetics. 2021 Mar 25;13:64. doi: 10.1186/s13148-021-01042-4 (PMC7995693; doi:10.1186/s13148-021-01042-4)
Supplement: Supplementary file 2 — Additional file 2. This additional file contains the comparison of ZBMIs between IoW and ALSPAC cohort. [file 13148_2021_1042_MOESM2_ESM.docx]

Supplemental Table 1: Comparison subsamples between the IoW and ALSPAC cohorts. In the following, one sample t-tests were applied for continuous variables, one sample proportion tests for percentages, and Chi-square tests for categorical variables.

|  | Subset sample  (n=325) * | ALSPAC  (n=713) | P value |
| --- | --- | --- | --- |
|  | **ZBMI (Mean ± SD)** | |  |
| **Pre-adolescence***** | 0.45 (1.04) | 0.28 (0.88) | <.0001 |
| Male | 0.43 (1.01) | 0.30 (0.88) | 0.004 |
| Female | 0.49 (1.09) | 0.27 (0.88) | <.0001 |
| **Post-adolescence***** | 0.53 (1.17) | 0.54 (0.87) | 0.70 |
| Male | 0.41 (1.16) | 0.56 (0.89) | 0.002 |
| Female | 0.69 (1.18) | 0.54 (0.87) | 0.0004 |
|  | **N (%)** | |  |
| **Gender** |  |  |  |
| Male | 186 (57.2%) | 368 (57.2%) | 0.003 |
| Female | 139 (42.8%) | 345 (42.8%) |  |
| **BMI Transition** |  |  |  |
| Normal 🡪 OwO** | 31 (10.8%) | 67 (9.4%) | 0.28 |
| OwO 🡪 Normal | 24 (8.3%) | 40 (5.6%) |  |
| OwO 🡪 OwO | 30 (20.4%) | 66 (9.3%) |  |
| Normal 🡪 Normal | 203 (70.5%) | 540 (75.7%) |  |
| Note: *Both subset samples in the IoW and ALSPAC cohorts are selected based on availability of DNA methylation data. ** OwO: Overweight/Obese.  *** Pre-adolescence: Age 10 in the IoW cohort and age 7 in the ALSPAC cohort. Post-adolescence: Age 18 in the IoW cohort and age 15 or 17 in the ALSPAC cohort. | | | |
